# Transcriptome Profiling in Human Diseases: New Advances and Perspectives

**DOI:** 10.3390/ijms18081652

**Published:** 2017-07-29

**Authors:** Amelia Casamassimi, Antonio Federico, Monica Rienzo, Sabrina Esposito, Alfredo Ciccodicola

**Affiliations:** 1 Department of Biochemistry, Biophysics and General Pathology, University of Campania “Luigi Vanvitelli”, Via L. De Crecchio, 80138 Naples, Italy; amelia.casamassimi@unicampania.it; 2 Institute of Genetics and Biophysics “Adriano Buzzati Traverso”, CNR, 80131 Naples, Italy; antonio.federico@igb.cnr.it; 3 Department of Science and Technology, University of Naples “Parthenope”, 80143 Naples, Italy; 4 Department of Environmental, Biological, and Pharmaceutical Sciences and Technologies, University of Campania “Luigi Vanvitelli”, 81100 Caserta, Italy; monica.rienzo@unicampania.it (M.R.); sabrina.esposito@unicampania.it (S.E.)

**Keywords:** transcriptome profiling, RNA sequencing, alternative transcripts, noncoding RNA, multi-omics, human diseases, biomarkers, therapeutic targets

## Abstract

In the last decades, transcriptome profiling has been one of the most utilized approaches to investigate human diseases at the molecular level. Through expression studies, many molecular biomarkers and therapeutic targets have been found for several human pathologies. This number is continuously increasing thanks to total RNA sequencing. Indeed, this new technology has completely revolutionized transcriptome analysis allowing the quantification of gene expression levels and allele-specific expression in a single experiment, as well as to identify novel genes, splice isoforms, fusion transcripts, and to investigate the world of non-coding RNA at an unprecedented level. RNA sequencing has also been employed in important projects, like ENCODE (Encyclopedia of the regulatory elements) and TCGA (The Cancer Genome Atlas), to provide a snapshot of the transcriptome of dozens of cell lines and thousands of primary tumor specimens. Moreover, these studies have also paved the way to the development of data integration approaches in order to facilitate management and analysis of data and to identify novel disease markers and molecular targets to use in the clinics. In this scenario, several ongoing clinical trials utilize transcriptome profiling through RNA sequencing strategies as an important instrument in the diagnosis of numerous human pathologies.

## 1. Introduction

Since before the completion of the Human Genome Project [1,2,3], transcriptomics has been a reference research field, especially in the study of human diseases [4]. Transcriptome contains the full information about all RNA transcribed by the genome in a specific tissue or cell type, at a particular developmental stage, and under a certain physiological or pathological condition [5,6]. Thus, transcriptome analysis not only allows us an understanding of the human genome at the transcription level, but also provides a comprehension of gene structure and function, gene expression regulation and genome plasticity. More importantly, it may disclose the key alterations of biological processes triggering human diseases, thus offering novel instruments useful not only for the comprehension of their underlying mechanisms but also for their molecular diagnosis and clinical therapy.

**Figure 1:**
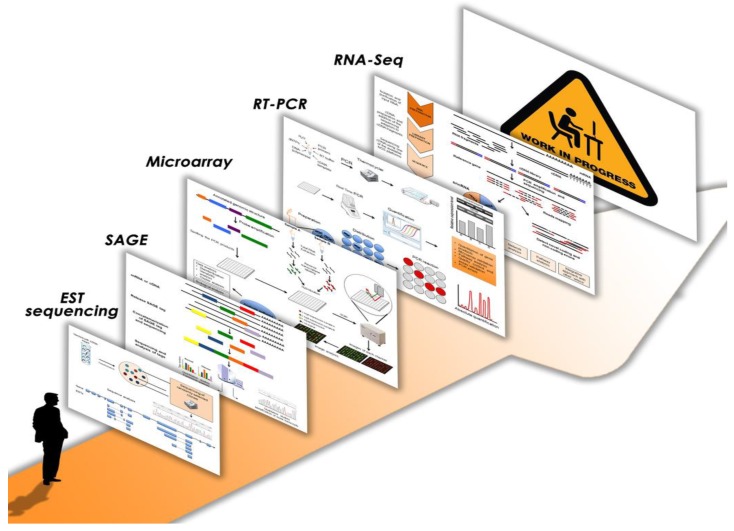
Transcriptomics: from the beginning to the most recent strategies and the road ahead. The single schemes are available as supplementary material.

The analysis of differentially expressed genes as a transcriptional response of the genome to different environmental stimuli or physiological/pathological conditions has always been one of the main purposes of transcriptome studies [4,5,6]. Figure 1 illustrates the most important progresses and innovations of the last years in this research field. The first approach to profiling human transcriptomes started with the publication of a database with human ESTs (expressed sequence tags), short sequences of cDNA clones obtained by the first DNA sequencers [7]. Subsequently, other methods, like SAGE (serial analysis of gene expression) and microarray, employing complementary probe hybridization, tried to quantify gene expression on a global basis [8,9]. Many important differentially expressed genes belonging to key molecular pathways were identified in several human pathologies through these strategies; particularly, the application of microarray technology proved to be valuable and became the most used for transcription profiling in the subsequent years, even though initially it showed some drawbacks regarding quantification [10,11,12,13,14,15]. Indeed, in the microarray procedure, the levels of hybridization are quantified using fluorescence that is converted into expression measurements. Since the fluorescent readout of hybridization intensities may change between different laser scanners, this resulted in low reproducibility between different laboratories. This issue was later overcome with the introduction of the MicroArray Quality Control consortium, which led to the development of quality control standards to establish a framework for the use of microarrays in both clinical and experimental settings [16]. Contextually, the quantitative reverse transcription PCR (qRT-PCR) method was often applied to validate the results from high-throughput platforms [17]. Indeed, this technique is considered the gold standard system for measuring transcript levels since it is fast, reliable, reproducible, sensitive and accurate, even though it is able to analyze one or a few genes in a single assay and could provide incomplete or misleading data if alternative splicing isoforms are present; in these cases proper primer design becomes a very critical step in the validation procedure [18,19]. Interestingly, in clinical settings, several qRT-PCR assays have been also developed for diagnosis and prognosis, treatment monitoring, pathogen detection and transplant biology [6,20,21]. Prospectively, the emerging method of digital polymerase chain reaction (dPCR) has the potential to be the new, future gold standard since it allows better quantification of the levels of all nucleic acids, including transcripts. Indeed, several commercialized dPCR apparatuses are already available and some other devices are under development; they are based on different principles of sample dispersion, amplification, and quantification and they enable absolute quantification of target microRNA (miRNA) as well as RNA sequencing (RNA-Seq) validation [22]. Additionally, despite some concerns about reproducibility, various microarray-based assays measuring several RNA targets at one time have also become clinically available. These multigene panels have wide-ranging clinical application and are especially utilized as a diagnostic support in oncology, in combination with clinic-pathological factors, to predict disease recurrence and response to different treatments, as well as to recognize cancers of unknown primary [6,23,24,25].

## 2. Novel Insights from Transcriptome Studies by Next Generation Sequencing

More recently, with the advent of next generation sequencing (NGS), microarrays have been progressively displaced by NGS-based RNA-Seq as the technology of choice for gene expression analysis. Noticeably, at the beginning of the NGS era, with the introduction of the first pioneer sequencing instruments, microarrays remained the favorite choice for most scientists. Indeed, these platforms, like 454/Roche pyrosequencing technology and Illumina/Solexa, exhibited some concerns, such as relatively high error rates or short reads, respectively [26,27]. Later, as soon as these platforms have been improved and the relative issues have been solved, NGS will have brought a significant qualitative and quantitative advance to transcriptome analysis that can be studied more quickly and at higher resolution. The first massive-scale RNA-Seq analysis of a non-ribosomal transcriptome in human diseases was performed on a well-studied human chromosome imbalance, the trisomy of chromosome 21 (Down Syndrome) by our group [28].

An important advantage of this technology is the possibility of also detecting and quantifying low-expressed genes that could not be revealed by microarray analysis [29,30,31]. Nevertheless, RNA-Seq provides more information on post-transcriptional RNA editing, especially splicing, since it is able to examine both known splice junctions and to discover novel splicing events [32]. Of note, to date the most used technology in transcriptome studies (Illumina), because of the short read length, does not allow linking directly to alternative exons that are separated by long distances. Thus, the reconstruction of full-length transcripts and the quantification of alternatively spliced isoforms requires the use of specific algorithms, which are continuously advancing [33]. Besides, further validation with additional methods are also warranted, thereby rendering the analysis of differential splicing not yet of routine use. Interestingly, new technologies, such as PacBio and Oxford Nanopore sequencing, have been recently developed which could potentially allow the study of full-length mRNA without assembly since they provide longer reads of several Kb, potentially covering full-length transcripts even though these methods currently exhibit high costs and error rates that prevent their application on a large scale [34,35]. A relationship between defective alternative splicing and human pathologies was already demonstrated through sequencing analysis many years ago, when our group also discovered a new exon in the *RPGR* (retinitis pigmentosa GTPase regulator) gene, preferentially expressed in mouse and bovine retina, which was mutated in patients with X-linked Retinitis pigmentosa [36]. Later, we also found an alternative transcript of the *PPARG* (peroxisome proliferator activated receptor gamma) gene that was overexpressed in colon cancer [37]. Nowadays, thanks to the RNA-Seq studies the link between splicing deregulation and many human diseases is continuously increasing, so that also therapeutic strategies specifically targeting splicing defects are currently under investigation [38,39]. Human diseases showing aberrant RNA splicing range from neurological pathologies to immunohematology disorders and malignancies [38]. For instance, a recent transcriptome sequencing study revealed aberrant alternative splicing in Huntington’s disease through the identification of 593 differential alternative splicing events between pathological and control brains [40]. Similarly, potential alternatively spliced RNA isoforms with psoriasis-specific expression profiles were also identified by RNA-Seq [41]. Besides, in cancer the splicing process is frequently disrupted thus appearing as one of the hallmarks of cancer; indeed, many cancer-specific splicing events, which are likely to contribute to disease progression, have been recently identified as the result either of mutations in splicing-regulatory elements or changes in components of the splicing machinery [42,43]. Moreover, recent literature provides evidence for epigenetic regulation of alternative splicing, even though the complex interplay between these two molecular mechanisms in many disease models is still poorly understood [44]. Thus, there is a need for future work on integrative analysis to understand the interplay between epigenetic modifications and aberrant splicing as well as between alterations of splicing transcripts and factors. As an example, recent integrated bioinformatics analysis of alternative splicing profiles in 491 lung adenocarcinoma (LUAD) and 471 lung squamous cell carcinoma (LUSC) patients, using RNA-Seq data in TCGA, showed different interactions between splicing factors and alternative splicing events in LUAD and LUSC prognostic models and splicing networks [45].

Moreover, RNA-Seq allows the analysis of allele-specific expression and the detection of fusion transcripts, which often occur in pathologies like cancer [6,30,31,46]. In addition, methodological innovations have overcome some limits of this technique. For instance, a series of methods associating RNA-Seq with cap analysis of gene expression or with a nuclear run-on assay have provided data on the rates of transcription initiation and elongation, as well as on the RNA polymerase pausing positions [28,31,47]. It is noteworthy that strategies linked to RNA isolation (such as ribodepletion, small- and miRNA isolation and purification) allow the choosing of specific RNA species before RNA-Seq experiments, thus expanding the level of transcriptome analysis [28,31,47]. Indeed, it is well known that besides the protein-coding mRNA, there is a great variety of non-coding RNA (ncRNA), which may have either structural roles or important functions in gene regulation [48,49,50]. Transcriptomics studies have shown that more than 93% of the human genome is transcribed into RNA but only 2% into mRNA, whereas the remaining percentage consists of ncRNA, which are mostly represented by rRNA but also include other important RNA species [30,31,48]. These RNA molecules can be classified based on their functions, into housekeeping and regulatory ncRNA. The first category includes those with structural and catalytic roles, including tRNA and rRNA involved in translation, small nuclear RNA (snRNA) and small nucleolar RNA (snoRNA) controlling mRNA and rRNA splicing respectively, guide RNA (gRNA) functioning in RNA editing, etc. [48,49,50]. Depending on their length, the regulatory group of ncRNA can be divided into short ncRNA, such as miRNA, short interfering RNA (siRNA) and Piwi-interacting RNA (piRNA), and long ncRNA (lncRNA). Due to their capability of inhibiting gene expression by translational repression or mRNA degradation, miRNA may play a crucial regulatory role in many biological processes, such as development, stress response, and cell behavior [51,52]. Instead, siRNA and piRNA mainly act in the gene silencing of transposons and repetitive sequences to maintain genomic stability [53,54,55].

Undoubtedly, in the last years the discovery of miRNA has revolutionized molecular cell biology studies, including transcriptomics, and consequently clinical approaches to human diseases. Mature miRNA are transcripts with 19–24 nucleotides that are usually evolutionarily conserved [56]. They can originate within introns or exons of genes, or in intergenic regions. Their biogenesis involves many molecular factors and steps beginning in the nucleus with the transcription of long precursor miRNA (pri-miRNA) of about 1000–3000 nucleotides by RNA polymerase II, usually cropped into a shorter miRNA precursor (pre-miRNA) of 60–100 nucleotides in length; these precursors are then transported through Exportin-5 to the cytoplasm where they are further processed into short miRNA duplex by Dicer and finally into the functional mature miRNAs that are incorporated in the RNA-induced silencing complex (RISC), which plays a crucial role in miRNA activity. Indeed, according to the general mechanism, this complex guides miRNA to target the 3′-untranslated regions of specific mRNA to ultimately inhibit protein synthesis by either translational repression or messenger degradation [56]. To the present time, the miRBase database (release 21) has catalogued about 28,000 mature miRNA sequences and more than 2500 in humans [57]. Since they regulate a vast number of mRNA targets, which are involved in numerous processes of cell physiology, it is unsurprising that their deregulation, which can occur through diverse mechanisms, has detrimental effects on normal cell functioning. The pathogenic role of miRNA was first reported in chronic lymphocytic leukemia where the miRNA cluster, miR-15a/16–1, is frequently lost, thereby acting as a tumor suppressor [58]. Later, many transcriptome studies have shown that miRNA are aberrantly expressed both in various human cancers and other diseases like cardiological and neurological ones [56,59,60]. Moreover, since miRNA expression profiling is able to differentiate between normal and pathological states they could be used as novel biomarkers in the diagnosis and prognosis of several human diseases.

Differently, lncRNAs, which are RNA polymerase II transcripts with a length > 200 nucleotides, are poorly conserved and lack an open reading frame [61,62]. They can be sense, antisense, intergenic, bidirectional, and intronic transcripts, and can also originate from the regulatory regions of other functional units like enhancer RNA [63], showing a great diversity in their biogenesis; additionally, most of them are expressed at low levels thus precluding their detection before the advance of RNA-Seq technologies [61,62]. These transcripts include diverse RNA classes with distinct functions that can exert through different mechanisms of actions, not yet fully characterized [61,62]. Basically, lncRNAs are known to participate to the organization of nuclear sub-structures and they may regulate protein-coding gene expression either positively or negatively, thus playing a basic role in many biological processes. Indeed, they may directly interact with RNA like miRNA, or alternatively, they may influence transcription indirectly by recruiting and/or interacting with other regulatory proteins or complexes, like RNA binding proteins, as well as with nucleosome remodeling factors. Moreover, they are also able to affect mRNA stability, thus acting at the posttranscriptional level [61,62]. Tens of thousands of lncRNA have been identified so far and annotated in public databases [64,65], where many others are expected to be added in the near future, especially through the use of novel strategies, such as targeted RNA-Seq (i.e., RNA CaptureSeq), which has been recently developed to reveal and quantify rare transcripts [66]. Besides, since many lncRNAs are antisense transcripts, the construction of directional libraries has allowed their better detection and discrimination from mRNA; so far, many different methods have been developed to preserve the orientation of the original RNA in the final sequencing library, and to facilitate strand-specific analysis of the resulting data [67,68]. As for the other ncRNA, even the deregulation of numerous lncRNA has been observed in many human diseases, from cancer to cardiac pathologies or neurodegenerative disorders [59,60,64]. For instance, among the others, the well-known *MALAT1*, one of the first lncRNAs to be associated with a human disease, was initially described as metastasis associated in lung adenocarcinoma transcript; then further studies demonstrated that it is involved also in other disorders such as diabetes complications, since its expression was found to be augmented in ischemic limbs [60]. Another example is β-site amyloid precursor protein cleaving enzyme-1 antisense transcript (*BACE1-AS*), whose deregulation is likely to initiate a cascade of events that lead to Alzheimer and other neurodegenerative diseases [59]. Of note, a high percentage of the identified lncRNA overlapped disease-associated single nucleotide polymorphisms (SNPs) [64,65]. Interestingly, a recent study, which demonstrates that *lnc13* is associated with susceptibility to an intestinal autoimmune disorder (i.e., celiac disease), also provides an important example of how the function of lncRNA can be directly affected by a disease-associated SNP [69].

More recently, circular RNA (circRNA), a novel class of lncRNA, have been highlighted; they are characterized by the presence of a covalent link between the 3′ and 5′ ends of protein-coding exons generated by back-splicing [70,71]. They are considered as active participants in the regulation of gene expression since they are likely to act essentially as cytoplasmic miRNA sponges and RNA-binding protein sequestering agents even though other mechanisms are now emerging [70,71,72]. Indeed, one of the best-characterized circRNA is ciRS-7 (from circular RNA sponge for miR-7), which is known to act as a sponge of miR-7 and to inhibit its activity resulting in increased levels of miR-7 targets [72]. Interestingly, ciRS-7 is located in the cytoplasm and contains more than 70 selectively conserved miRNA target sites; moreover, it is also able to associate with the miRNA effector protein Argonaute in a miR-7-dependent manner [72]. However, the same circRNA has been newly defined to function even through regulation of the β-site APP-cleaving enzyme 1 (BACE1) and the β-amyloid precursor protein (APP) protein levels by promoting their degradation via the proteasome and lysosome; consequently, the level of β-amyloid peptide (Aβ) was reduced, suggesting that ciRS-7 may play a role in the protection of neuronal cells [73]. Furthermore, a very recent paper has demonstrated for the first time that another circRNA specifically controlling myoblast proliferation, circ-ZNF609, can be also translated into a protein thus highlighting a further mechanism of action for this class of RNA [74]. The lack of an open end prevents RNA degradation by conventional pathways like RNase R and this resistance make circRNA extraordinarily stable RNA molecules, thus suggesting they could be utilized as a novel class of biomarkers [75]. Currently, thousands of human circRNA have been identified through molecular biology strategies combined with bioinformatics approaches and have been collected in public online databases. Remarkably, high-throughput sequencing analyses indicate that most circRNA exhibits cell-, tissue- and developmental stage-specific expression, suggesting that they may play crucial roles in multiple cellular processes [70]. Thus, it is not surprising that the deregulation of many circRNA has been found to have relevance in many human diseases, including cancer, cardiovascular, neurological and developmental disorders, among others [76,77]. Of note, the ciRS-7/miR-7 axis is fundamental for many biological processes and it has been involved in several pathologies, especially in cancer, where it plays an essential regulatory role in cancer-associated pathways [76,77]. Similarly, other circRNA-miRNA axes, such as the circ-Sry/miR-138 axis, are also deregulated in cancer [76,77].

Despite all the advantages of RNA-Seq compared to hybridization-based techniques, there are also several challenges related to the management and the interpretation of data. Indeed, although the main analytical steps of the analysis are unchanged, the computational pipeline should be adapted to each experimental design, organism studied and research goals [33]. Moreover, many experimental parameters and strategies should be set in order to plan the sequencing experiments to answer the underlying biological questions. For instance, the statistical power and the sample size should be determined while setting the experimental plan in order to obtain reliable results, especially in experiments with multiple conditions and outcomes, where a fraction of the results may be erroneously considered as significant [78]. This sheds light on the need for biological and/or technical replicates in order to control unwanted technical or biological variability affecting the sample preparations. Another parameter that should be considered is the desired sequencing coverage depth, which refers to the average number of reads mapping on the reference transcriptome (or genome). Such a parameter can differ on the basis of the application and the aim of the work. Essentially, higher coverage allows the detection of rarely expressed transcripts, novel splice junctions and 3′ UTRs and new expressed intergenic regions, whereas lower coverage permits only reporting on the highly expressed transcripts [79]. Furthermore, from the computational point of view, the choice of the appropriate algorithm for preprocessing, mapping, filtering, normalization and differential expressed gene detection is quite challenging and still under evaluation. Indeed, to date a plethora of freely available software has been developed, each one showing specific advantages and weaknesses. For instance, Engstrom and colleagues showed a detailed comparison of the performance of the main mapping algorithms [80]. Comprehensive studies comparing the most utilized methods for read-count normalization and the identification of significantly deregulated genes between two or more conditions are well described elsewhere [81,82].

## 3. Transcriptome Studies by NGS: the Work in Progress

One of the restraints of standard transcriptome studies is the requirement of considerable amounts of cells/tissues to get a valid gene expression profile, since often, under certain conditions, only a small extent of material is available. Moreover, a general feature of biological tissues is cell heterogeneity, which assumes great importance especially in the study of particular disorders. Specifically, the identification of distinct phenotypic cell types within a heterogeneous population, together with their molecular investigation, can be applied to many research fields ranging from embryonic development and stem cell differentiation to immunology and oncology. For instance, a very recent study using the unbiased single-cell RNA-Seq of ~2400 cells has revealed new types of human blood dendritic cells, monocytes and circulating progenitors, thus providing a revised taxonomy, which will support immune monitoring in both health and disease [83]. Thus, single-cell RNA-Seq represents the new frontier in transcriptome profiling as well as in other omics studies [84]. However, more sensitive techniques are needed to analyze transcriptomes at the single-cell level. To this purpose, several technologies have been developed promptly in the last years, including a variety of cDNA amplification methods and new computational and statistical models and tools [47,84,85,86,87].

In the NGS era, the continuous production of large datasets from transcriptomics and other omics projects has prompted the birth of big consortia with the aim to collect, share and make available these multidimensional data to the scientific community. Mostly, this has been realized in cancer research due to the easy achievability of tumor tissue biopsies. Indeed, clinical and omics data for thousands of patients are accessible at the Cancer Genome Atlas (TCGA) [88] or at the International Cancer Genome Consortium (ICGC) [89]. Nevertheless, although with lower numbers of samples, multi-omics research has extended also into other research fields such as brain diseases with the Allen Human Brain Atlas [90], which also includes RNA-Seq datasets from the Aging, Dementia and Traumatic Brain Injury Study. Other valuable databases include the Gene Expression Omnibus (GEO), [91], ENCODE [92] and the Genotype-Tissue Expression (GTEx) Project [93], among others. These web resources contain expression data from many human normal cell lines and tissues.

The availability of huge amounts of data, especially in the cancer research field, has stimulated many transcriptome studies, even focused on the analyses of specific biochemical pathways, molecular complexes or gene families either in pan-cancer or definite tumor types [94]. Moreover, TCGA or GEO datasets have been also very useful to validate results from independent small cohorts of patients [95]. More importantly, they have encouraged the search for novel methodological and computational approaches to investigate cancer regulatory networks, as detailed below [96].

## 4. Integrated Omic Analysis: Beyond Transcriptomics

The huge advances in the development of new, high throughput sequencing approaches, not only in transcriptomics, but also in genomics, epigenomics, proteomics and metabolomics, have increased the complexity of the analytical methods aimed at the identification of the molecular basis of phenotypic traits, especially in complex diseases. In particular, through the advent of novel “omic” technologies and longitudinal studies performed on a large scale by big consortia, biological systems are investigated at an unprecedented scale, generating a large and often heterogeneous amount of data [97]. Recently, in order to facilitate the management and analysis of data and to identify novel biomarkers in human diseases, multi-omic data integration approaches have been developed. Until recently, data integration was small in scale and limited to two layers, mostly because of the lack of data across multiple layers for the same experimental conditions. Notably, among the used approaches, a research group developed and implemented two affordable methods to integrate and select variables deriving from two different types of omics. Specifically, they advanced an R package called “ntegrOmics” based on the regularization of the canonical correlation analysis (CCA) in cases where *p >> n*, and of the sparse partial least square regression (PLS), consisting of a variant of the classical PLS [98]. Importantly, this vertical and horizontal integration will become more effective and meaningful as more data are added within and across multiple layers. Multiple layer integration could lead to lower false discovery rates and an improved portrait of cellular systems. Despite these advantages, analyzing multiple datasets deriving from different platforms and experimental procedures is somehow challenging since systematic biases exist due to technological platforms, laboratories and analysis methods. Several methods have been developed in order to overcome the complexity related to the integration of more than two omics and to remove the existing bias caused by technical differences as well as the batch effect. Indeed, these discrepancies could create obstacles for the application of machine learning and modeling techniques, which aim to learn from data. A recent overview focuses on mathematical and methodological aspects of the most common existing methods developed for multi-omic data analysis [99]. The authors considered two main criteria for categorizing them: the network-based methods, if they are based on the graphs to model the interactions among the variables, and the Bayesian methods, if they allow the computing of the posterior probability distribution making use of the Bayes’ rule. Moreover, they also evaluated the specificity of methods in order to assess whether a certain method is able to analyze only two (or more) specific omics, such as Conexic [100] or they can analyze any of the combinations, as iCluster [101]. Despite the existing methods allowing researchers to extract affordable information from the integration of multiple omic layers, useful for the prioritization of variable and their interactions for in vitro experiments, these approaches often lack visualization outputs to fully unravel the complex associations between different biological entities as, for example, in the case of physiological and pathological states [102]. Noteworthy, the “mixOmics” R package, was originally developed to provide several graphic outputs useful to extract meaningful information upon the application of multi-omics integrative approaches, including correlation circle plots, relevance networks and clustered image maps (also known as heatmaps) [102]. The correlation circle plot has been widely used to graphically visualize the relationship among samples through principal component analysis (PCA) in one-layer omic analyses. The use of such a graphical tool needs a generalization in order to represent variables deriving from two different datasets using the aforementioned statistical approaches, such as CCA and partial least squares regression. Relevance networks are one of the most intuitive graphics used to identify binary interactions between biological entities, such as pathway interactions. This method generates an interaction map where the nodes are the variables and the edges indicate the type of relationship between the nodes. Generally, the represented variables are of the same type, whereas in the case of multi-layer analyses performed by mixOmics they can be derived by two different platforms and the network is inferred on the basis of the results of regularized integrative approaches. The clustered image map is the most diffused large-scale data visualization method. Usually, it is used in transcriptomics to show the gene expression profiles among different conditions. In the cases of pairwise omics integration it can be helpful to characterize the Pearson correlation between the two datasets. Moreover, it is very powerful to perform a hierarchical cluster of the variables or on the samples in order to identify the biologically meaningful subset of correlated variables [102]. Despite the existing statistical methods being mainly addressed to NGS data analysis and management, current research needs to develop and improve multi-layer data integrative methods for multi-omic derived data. Although multi-omics research is still challenging, it will accelerate the new discoveries and insights into human diseases in the near future. Indeed, it will allow their early diagnosis and will help clinicians and families to forecast and make informed decisions about the prognosis and, prospectively, will provide tailored therapies [103].

## 5. Conclusions

In the last years, transcriptome analysis has been completely revolutionized, formerly with the adoption of microarrays and successively with the introduction and implementation of NGS platforms for RNA sequencing. Particularly, RNA-Seq has the ability to simultaneously detect whole gene expression levels and the diverse species of the RNA world; the availability of such a complete transcriptome profile has been a powerful tool to obtain insights into the molecular mechanisms underlying human pathologies and could be very useful prospectively in clinical testing for a wide range of diseases. Indeed, many clinical trials are now utilizing molecular profiling in order to better stratify patients for their access to new therapies with targeted agents, especially in the care of human malignancies [104]. To date, several strategies have been used to measure clinically relevant RNA species in various human diseases and many others are under investigation (Figure 1). Particularly, recent efforts are now ongoing to better standardize RNA-Seq accuracy and reproducibility as well as sensitivity, specificity and precision in all the steps of this technique in order to expand the clinical utility of transcriptome profiling in human diseases [6]. Moreover, since the enormous amount of data produced by RNA-Seq might restrain its use in molecular diagnosis, a targeted RNA-Seq method could represent a better approach because of its reliability, feasibility and also minor costs. Indeed, this technology offers the option to use both ready-to-go commercially available gene panels and custom panels. As an example, AmpliSeq-RNA panels are constructed for the study of a small number of pre-defined gene sets (from 150 to 900 genes) [105,106]. Importantly, the explosion of this approach has also required a huge effort in the bioinformatics field to find the best analysis pipeline. Nowadays, the further integration of transcriptomics with other omic strategies, together with single cell omics, will provide a more complete understanding of how single cells and different tissue types are organized and controlled and how they are altered in a specific pathological state, thus offering the opportunity of tailored therapeutics.

## Abbreviations

CCA	Canonical Correlation Analysis
circRNAs	Circular RNAs
dPCR	Digital Polymerase Chain Reaction
ENCODE	Encyclopedia of the regulatory elements
ESTs	Expressed Sequence Tags
GEO	Gene Expression Omnibus
gRNAs	Guide RNAs
GTEx	Genotype-Tissue Expression
ICGC	International Cancer Genome Consortium
lncRNAs	Long ncRNAs
LUAD	Lung adenocarcinoma
LUSC	Lung squamous cell carcinoma
miRNAs	microRNAs
ncRNAs	Non-coding RNAs
NGS	Next Generation Sequencing
PCA	Principal Component Analysis
piRNAs	Piwi-interacting RNAs
PLS	Partial Least Square
qRT-PCR	Quantitative Reverse Transcription PCR
RISC	RNA-induced silencing complex
RNA-Seq	RNA-Sequencing
SAGE	Serial Analysis of Gene Expression
siRNAs	Short interfering RNAs
snoRNAs	Small nucleolar RNAs
snRNAs	Small nuclear RNAs
TCGA	The Cancer Genome Atlas
